# Mental Health in Multiple Sclerosis Patients without Limitation of Physical Function: The Role of Physical Activity

**DOI:** 10.3390/ijms160714901

**Published:** 2015-07-02

**Authors:** Alexander Tallner, Anne Waschbisch, Christian Hentschke, Klaus Pfeifer, Mathias Mäurer

**Affiliations:** 1 Institute of Sport Science and Sport, University of Erlangen-Nürnberg, Gebbertstr. 123b, 91058 Erlangen, Germany; E-Mails: alexander.tallner@fau.de (A.T.); christian.hentschke@fau.de (C.H.); klaus.pfeifer@fau.de (K.P.); 2 Department of Neurology, University of Erlangen-Nürnberg, Schwabachanlage 6, 91054 Erlangen, Germany; E-Mail: Anne.Waschbisch@uk-erlangen.de; 3 Caritas Krankenhaus Bad Mergentheim gGmbH, Uhlandstr. 7, 97980 Bad Mergentheim, Germany

**Keywords:** multiple sclerosis, physical activity, quality of life, depression, mobility limitation, mental health

## Abstract

Multiple sclerosis (MS) patients, in general, show reduced physical function, physical activity, and quality of life. Positive associations between physical activity and quality of life have been reported. In particular, we were interested in the relation between physical activity and mental health in MS patients without limitation of physical function, since limitations of physical function may influence both physical activity and quality of life. Assessment comprised the Baecke questionnaire on physical activity, the Short Form 36 Health Survey (SF-36), and Beck Depression Inventory (BDI). We ranked our sample according to physical activity into four groups and performed an ANOVA to analyze the relationship between levels of physical activity and health-related quality of life (HRQoL). Then we performed a subgroup analysis and included patients with unlimited walking distance and a score of less than 18 in the BDI. Most active *vs*. inactive patients were compared for the mental subscales of the SF-36 and depression scores. From 632 patients, 265 met inclusion criteria and hence quartiles were filled with 67 patients each. Active and inactive patients did not differ considerably in physical function. In contrast, mental subscales of the SF-36 were higher in active patients. Remarkable and significant differences were found regarding vitality, general health perception, social functioning and mental health, all in favor of physically active patients. Our study showed that higher physical activity is still associated with higher mental health scores even if limitations of physical function are accounted for. Therefore, we believe that physical activity and exercise have considerable health benefits for MS patients.

## 1. Introduction

Multiple sclerosis (MS) is a chronic inflammatory disease of the central nervous system that mainly affects young adults [1]. Brain inflammation often leads to functional impairments and reduced mobility, which is associated with both low levels of physical activity and reduced health-related quality of life (HRQoL) compared to healthy controls [2,3]. Physical inactivity and the resulting symptoms and comorbidities have the potential to further decrease health status and HRQoL in MS [4].

It has been shown that exercise has the capability to alleviate MS-specific symptoms and functional deficits. Exercise has proven effects on walking ability [5] and on muscle strength and aerobic capacity [6,7]. Research so far has focused on the relation between exercise and physical function, rather than mental health.

The World Health Organization defines mental health as “a state of well-being in which the individual realizes his or her own abilities, can cope with the normal stresses of life, can work productively and fruitfully, and is able to make a contribution to his or her community” [8]. However, MS patients often have difficulties coping with stress and report considerable limitations concerning social relationships [9]. Hence, quality of life in MS patients is generally worse than in the general population, and concerning disease course, it is lower in patients with progressive MS compared to patients with relapsing-remitting MS [10]. Since MS patients are not only concerned about physical disability but also about role limitations, social status, or emotional aspects, quality of life can dramatically worsen even at a point when physical disability is not yet a major problem [11].

Therefore, we were interested in the relation between physical activity and mental health in multiple sclerosis patients. In particular, we concentrated on patients without limitation of physical function, because in this particular group the influence of physical disability on quality of life can be excluded.

## 2. Results

We distributed 1640 questionnaires. 632 patients (38.5%, 460 female, 172 male) returned the questionnaires and were included for the analysis. Mean age was 43.3 ± 10.4 years, mean expanded disability status scale (EDSS) was 3.0 ± 1.8 and mean disease duration was 10.3 ± 7.8 years. Table 1 shows the HRQoL of our study sample characterized by the eight subscales of the Short Form 36 Health Survey (SF-36), compared with the corresponding values of German healthy controls [12]. According to a *t*-test, our study sample showed statistically significant lower values for all SF-36 subscales; effect sizes were moderate to large except for bodily pain, where only a small effect could be seen.

**Table 1:** Characteristics of the study sample and German healthy controls [12] concerning the SF-36 subscales (mean = mean value, SD = standard deviation).

	SF-36	Physical Function	Role Physical	Bodily Pain	General Health	Vitality	Social Function	Role Emotional	Mental Health
Study sample	mean	65.24	58.90	74.09	54.63	47.47	71.62	68.2	66.23
	SD	29.73	40.67	28.6	21.3	19.87	26.28	40.56	18.49
German healthy controls	mean	83.57	80.0	77.1	66.05	61.75	87.66	87.74	72.79
	SD	23.8	34.5	28.4	21.1	19.2	19.45	28.9	17.37
Group differences	significance	*p* < 0.001	*p* < 0.001	*p* = 0.014	*p* < 0.001	*p* < 0.001	*p* < 0.001	*p* < 0.001	*p* < 0.001
	Effect size (Cohen’s d)	0.68	0.59	0.11	0.54	0.74	0.77	0.62	0.37

**Figure 1:**
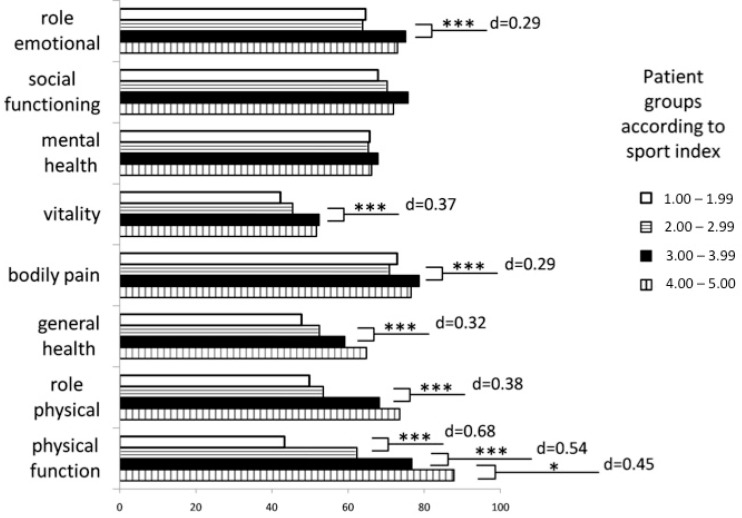
Sport activity and health-related quality of life (SF-36). Sport activity (sport index of the Baecke Questionnaire) and the SF-36 subscales with additional information on effect size (Cohen’s d) and level of significance between two adjacent patient groups (* *p <* 0.05, *** *p <* 0.001). Patients are grouped according to the sport index value, with higher values indicating higher sport activity.

Analysing the association between physical activity and HRQoL, the analysis of variance revealed significant overall differences between the groups according to the sport index for all but two subscales of the SF-36 (physical function *p* < 0.001, role physical *p* < 0.001, bodily pain *p* = 0.03, general health *p* < 0.001, vitality *p* < 0.001, social functioning *p* = 0.05, role emotional *p* = 0.017, mental health *p* = 0.525). *Post-hoc* tests and computed effect sizes showed that the association was more pronounced for the physical subscales of the SF-36, in particular physical function, compared to the mental subscales (Figure 1).

Because of the pronounced association between physical activity and physical function and the fact that both of them might be related to mental domains of HRQoL, we only included patients without physical limitations (indicated by an unlimited walking distance) or mood disorders (indicated by a sum score of less than 18 of the beck depression inventory (BDI) questionnaire) for further analysis of the net relation between physical activity and mental health.

265 patients fulfilled the inclusion criteria (68 male, 197 female; age 39.4 ± 9.2; EDSS 1.6 ± 0.9). We ranked all these patients according to the sport index of the Baecke Questionnaire, established Quartiles and compared the most (first quartile) and the least physically active patients (fourth quartile) with regard to psychological subscales of the SF-36. Table 2 shows the characteristics of these MS patients without physical disability.

**Figure 2:**
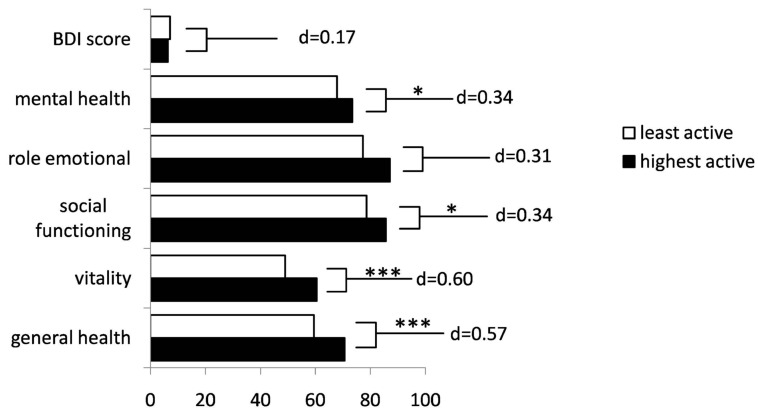
Comparison of physically active and inactive patients concerning health-related quality of life. Data taken from a subgroup of patients without limitation of walking distance and without depression. Bars show the psychological subscales of the SF-36 of the least and highest physically active quartile of the subgroup (*n* = 66 per quartile), with additional information on effect size (Cohen’s d) and level of significance (* *p <* 0.05, *** *p <* 0.001).

We could show that physically active patients report a significantly better status in all mental subscales of the SF-36 but emotional role function, where a trend was detected. Effect sizes were small to moderate in magnitude (Figure 2). No differences with respect to depression expressed by the sum score of the BDI could be recognized.

**Table 2:** Characteristics of the active and inactive multiple sclerosis (MS) patients without physical disability (mean = mean value, SD = standard deviation, EDSS = expanded disability status score, BMI= body mass index, BDI = beck depression inventory).

Activity	*N* (Male; Female)		EDSS	MS Duration	Age	BMI	Sport Index	Leisure Index	Work Index	BDI Score
Inactive	66 (m:16; f:50)	mean	1.8	7.3	37.8	23.3	2.1	3.1	2.5	7.1
		SD	1.0	6.0	10.4	4.0	0.4	0.9	0.7	4.4
Less active	66 (m:14; f52)	mean	1.6	8.7	40.2	23.6	2.8	3.3	2.4	7.1
		SD	0.9	7.4	10.1	4.3	0.1	0.8	0.6	4.5
Active	67 (m:11; f:56)	mean	1.8	7.7	39.3	23.5	3.3	3.5	2.4	7.0
		SD	1.0	6.4	8.4	3.5	0.2	0.8	0.6	4.4
Very active	66 (m:27; f:39)	mean	1.4	7.4	40.2	22.5	4.0	3.7	2.3	6.2
		SD	0.9	5.3	7.7	3.7	0.4	0.8	0.6	4.6

## 3. Discussion

Health-related quality of life (HRQoL) comprises physical, psychological and social domains of life quality that are influenced by health status [13].

Levels of HRQoL in our study sample were reduced compared to healthy controls, which is in line with other research [3,14]. Explanations for low HRQoL in MS patients are the unpredictable and progressive course of the disease, the absence of a cure for MS, inefficiency, and adverse effects of disease-modifying therapies and the disease onset in the most productive life span of young adults [14]. Physical disability, fatigue, depression, cognitive impairments, chronic pain as well as bladder, bowel, and sexual dysfunction have been shown to be negatively associated with HRQoL in MS. That impairments in mental functions and depression might be the strongest predictors of low HRQoL has shown that symptoms do not appear independently but, often having common etiology, build up symptom clusters [15,16,17,18,19]. Fatigue, depression, pain and cognitive disorders represent such a symptom cluster; and there appears to be a dose-response-relationship between the worsening of symptoms and the worsening of HRQoL. In a nutshell, HRQoL in MS patients is reduced predominantly owing to impairments in mental and physical function, caused by MS-specific symptoms, with the overall impact possibly being mediated by psychological variables like coping strategies and self-efficacy [9,20,21].

Consequently, interventions enhancing HRQoL in MS should target either physical functions like walking ability, strength, or endurance, neuropsychological symptoms like depression or psychological variables like self-efficacy. Therefore, physical activity and exercise might be key components of intervention since our study has clearly identified a meaningful association between the level of physical activity and health-related quality of life, indicated by significant group differences and moderate to large effect sizes.

Our results are in line with a recent meta-analysis by Motl and Gosney [2] showing that exercise is associated with an improvement in HRQoL of about one fourth standard deviation. According to the authors, this improvement is small but clinically relevant as it is comparable to effects of disease-modifying treatments concerning the reduction of exacerbations. In addition, a longitudinal study by Stuifbergen *et al.* [22] suggests that high levels of physical activity protect from ensuing loss of function, indicating that physical activity might help managing symptoms in MS.

However, one has to take into account that the relationship between physical activity and symptoms is bidirectional: symptoms may act as an antecedent or consequence of physical activity [23]. Therefore, higher levels of physical activity might be explained by fewer functional limitations, which facilitate participation in physical activity. Accordingly, the higher HRQoL scores of physically active MS patients in our study might also be caused by lower functional limitations.

Hence, we aimed to exclude the influence of physical function in order to extrapolate the net association between physical activity and HRQoL. This was the reason for a subgroup analysis of MS patients without limitation of walking ability and without depression according to the BDI. We focussed on the effect of physical activity on mental components of HRQoL and neglected physical components of HRQoL. We could clearly show that physical activity is associated with significantly better outcome in almost all mental components of the SF-36, with small to moderate effect sizes. This suggests that physical activity can be an aid to ameliorate mental and psychological aspects of MS.

For this subgroup analysis, we chose walking ability as distinctive variable to identify patients without limitation of physical function. This seems justified as walking ability can be seen as an indicator of disease progression and disability [4] and has been shown to be, among bodily functions, the most valuable function for patients, regardless of disease severity [24]. We relied on self-report data to be able to include a large study sample. Data quality may therefore be compromised. We assessed self-reported walking ability with the help of a single item question which was not validated psychometrically. This may be a drawback to the study. The same holds true for the EDSS, which was also only obtained via self-report and not assessed by physicians for this study. Nevertheless, patient ratings may be more closely linked to health-related quality of life than physician-rated measurements of impairment and disability [14]. In a previous analysis of the same sample, we could show, that self-reported physical activity in active and inactive subgroups corresponds nicely to objectively assessed aerobic capacity [25]. Therefore, the reliance upon self-report data may be justified.

The assessment of physical activity via questionnaire is not without pitfalls but considered appropriate in MS patients [5]. The psychometric properties of the Baecke-Questionnaire have been confirmed for the original version [26] and the German version [27] for non-diseased populations. In a recent validation study with 45 MS patients, we compared the German version to aerobic fitness assessed via spiroergometry and daily steps assessed via accelerometry [28]. Results showed significant and meaningful correlations of the sport index to daily steps (Pearson’s correlation coefficent *r* = 0.533, *p <* 0.001) and aerobic fitness (*r* = 0.487, *p =* 0.001). Thus, the German version of the Baecke Questionnaire is the only German questionnaire that has been evaluated for MS patients. In addition, the English version of the Baecke Questionnaire has shown sensitivity to change in exercise interventions with MS patients [29], which further justifies the choice of the questionnaire.

## 4. Experimental Section

The study was approved by the local ethics committee (Institutional Review Board University of Erlangen, December 2007). After written informed consent we collected questionnaires from patients with multiple sclerosis. Data included demographics, disease course, immunomodulatory treatment, and expanded disability status scale (EDSS). Where EDSS was not assessed, participants were asked to obtain their current EDSS-Score from their neurologist. In terms of ambulation we asked if walking distance is limited (via one item question) and to specify the limited walking distance in meters (one item). Physical activity, health-related quality of life and depression were evaluated using validated questionnaires.

### 4.1. Physical Activity

Habitual physical activity was assessed with the German version [30] of the Baecke questionnaire [31]. This instrument quantifies structured exercise (sport index) as well as physical activity in leisure time (leisure time index) and at work (occupational index). Each index adopts values from 1 to 5, with 5 indicating the highest possible physical activity. The questionnaire asks for habitual physical activity and does not specify a delineated period of time. Reliability and factor structure of the three indexes was confirmed for the German version [27,30,32].

### 4.2. Health-Related Quality of Life

We employed a generic questionnaire, the SF-36 [33], in the German version [12,34]. The SF-36 comprises 36 Items in eight subscales, each scale adopting values from 0 to 100, 100 indicating the highest possible quality of life. Four subscales each constitute a mental (MCS) and a physical component summary scale (PCS). The underlying factor structure for the calculation of the summary scales does not apply appropriately to MS-patients [35,36], so summary scales will not be computed.

### 4.3. Depression

Depression was assessed with the German version of the beck depression inventory (BDI) [37]. The BDI consists of 21 item groups which add to an overall sum score between 0 and 63. A sum score lower than 11 stands for no depression, 11–17 for a mild depression and values higher than 18 for moderate or severe depression.

### 4.4. Analysis and Statistics

Descriptive analyses were performed with the software SPSS Statistics, version 17.0 (IBM, Ehingen, Germany). We performed a complete case analysis; missing values were not imputed. We calculated the mean values and standard deviations of physical activity (sport index, leisure time index, occupational index) and quality of life (the four physical and four mental subscales of the SF-36). The authors of the German version of the SF-36 [12] provide the raw data of a German representative sample (*n* = 2914), which enabled us to perform a *t*-test and compute effect sizes (Cohen’s d) to quantify the size of difference between the study sample mean value and the representative sample mean value.

The sport index has, among the indexes of the Baecke questionnaire, the best predictive value for HRQoL [30]. Consequently, we classified the participants into 4 groups according to the sport index with upper cut-off values for groups set at 2.0, 3.0, 4.0 and 5.0, respectively. In a one way analysis of variance with *post-hoc*-tests (Bonferroni), we then compared those groups with respect to the physical and mental subscales of the SF-36. Cohen’s d was used to quantify the effect sizes for significant group differences.

In order to clarify the net influence of physical activity on psychological aspects of HRQoL, a potential influence of physical function on HRQoL has to be eliminated. Therefore, we included patients who reported an unlimited walking distance and who showed at most mild depression, corresponding to a score of less than 18 in the Beck Depression Inventory, in a subgroup analysis. We assumed that moderate or strong depression influences HRQoL considerably and therefore excluded those patients to avoid a bias.

We then ranked these patients according to the sport index of the Baecke Questionnaire, established quartiles and compared the highest (first quartile) and the least physically active patients (fourth quartile) with regard to the psychological subscales of the SF-36 with a *t*-test. Again, effect sizes were computed with Cohen’s d.

## 5. Conclusions

We could show that high levels of physical activity are associated with high levels of physical function (Figure 1). Interestingly, this association holds also true for patients in the highest sport index category (values between 4.0 and 5.0). Therefore, we assume that high levels of physical activity are not detrimental to physical function in MS patients without disability. A sport index value of 4.0 can be achieved, for example, with about six to eight weekly hours of moderate intensity exercise.

In addition to other studies showing beneficial effects of physical activity on various physical MS symptoms [5,38,39,40,41] our study gives evidence for a positive relationship between mental components like vitality, mental health, and emotional role function on the one hand and physical activity on the other hand. Therefore we believe that physical activity and exercise have a considerable health benefit for MS patients and, consequently, should represent an essential part of successful symptom management in MS therapy, especially in early disease stages. Physical activity could serve as a prophylactic treatment in order to maintain mental health and self-efficacy of otherwise healthy MS patients. In order to finally approve these associations, results from our cross-sectional study should be confirmed in a large prospective epidemiological study.
